# A one-year cohort study of complications, continuation, and failure rates of postpartum TCu380A in Tanzania

**DOI:** 10.1186/s12978-020-00999-4

**Published:** 2020-10-06

**Authors:** France John Rwegoshora, Projestine Selestine Muganyizi, Grasiana Festus Kimario, Ponsian Patrick Paul, Anita Makins

**Affiliations:** 1 Obstetrician & Gynecologist, Mbeya Zonal Referral Hospital, P.O.Box 419, Mbeya, Tanzania; 2 Department of Obstetrics & Gynecology, Muhimbili University of Health and Allied Sciences (MUHAS), P.O.Box 7623, Dar es Salaam, Tanzania; 3 FIGO-TAMA PPIUD project, P.O.Box 65222, Dar es Salaam, Tanzania; 4 Obstetrician/Gynecologist & Public Health, FIGO PPIUD Initiative, FIGO House Suite 3, Waterloo Court, 10 Theed Street, London, SE1 8ST UK

**Keywords:** Immediate postpartum, Contraception, Copper T380A, Complications, Tanzania

## Abstract

**Background:**

Less than 1% of married women in Tanzania use an Intrauterine Contraceptive Device (IUD) for contraception. An initiative by the International Federation of Gynecology and Obstetrics (FIGO) has been in progress since 2015 resulting in escalated method uptake in implementing hospitals. This study investigates failure rate, complications, and risk factors for one-year continuation of TCu380A IUD when used for immediate postpartum contraception under the initiative in Tanzania.

**Methodology:**

A prospective cohort study of women who had TCu380A insertion within 48 h of delivery in 6 hospitals in Tanzania between 1st December 2017 and 18th April 2018 was conducted. Face to face post insertion interviews were made with 1114 clients before discharge and later through phone calls up to the beginning of 13th month postpartum. Postpartum Intrauterine Device (PPIUD) continuation status, complications, duration of time they stayed with the IUD and the currently used method if PPIUD was discontinued were enquired. The outcome variable was PPIUD continuation at one year of IUD insertion. Data were analyzed using Statistical Product and Service Solutions software (SPSS) for Windows version 20 (IBM SPSS Statistics, Chicago, IL, USA).

**Results:**

In total 511(45.8%)clients had consented and availed to complete the one-year follow-up. Out of these, 440 still had IUD, giving a one-year continuation rate of 86.1%. Most (63%) IUD discontinuations occurred in the period between 7th week and 6 months of insertion. One-year method expulsion rate was 2.1%. There was one reported pregnancy that gives a method failure rate of about 2 per 1000. The independent risk factors in favor of method continuation at one year were absence of medical or social problem, being a youth (16–24 years), and delivery by Cesarean section.

**Conclusions:**

The continuation rate when CuT380A is used for immediate postpartum contraception is high, with low complication and failure rates. Some medical and social factors are important for method continuation, hence the need to consider in training, counselling and advocacy.

## Plain English summary

In Tanzania, less than one in a hundred women are utilizing an intrauterine contraception device (IUD) as a method of contraception. Particularly, the use of IUD immediately following delivery (referred to as the postpartum period) is extremely low despite its documented benefits. In order to mitigate this, since 2015 the International Federation of Gynecology and Obstetrics (FIGO) has been implementing a PPIUD initiative to institutionalize PPIUD as a routine service in antenatal and maternity ward settings. However, little is known about the long term safety, continuation and method effectiveness in a Tanzanian context where the acceptance of modern contraception is generally low.

In this study, 511 PPIUD clients in 6 hospitals under the FIGO initiative were enrolled during their antenatal attendances, face to face interviewed after PPIUD placement and later through phone calls at the beginning of their 13th month following the placement. PPIUD continuation status, complications, duration of time they stayed with the IUD and the currently used method if PPIUD was discontinued were enquired on the final interview.

We found a high continuation rate of the method after one year. Almost two thirds of those who discontinued the method did so during the time period between 7th week and 6 months of puerperium which should be regarded as critical. Spontaneous fall out of an IUD occurred rarely and only one pregnancy occurred within the year of follow-up which is equivalent to less than 2 pregnancies per 1000 clients. Absence of medical or social complaint, being in youth age group (16–24 years), and delivery by Cesarean section rather than the normal route were the outstanding reasons in favor of method continuation at one year.

These results affirm the safety, effectiveness and acceptance of PPIUD and underscore the need to integrate this information in health educational material, training of service providers, counselling and advocacy for PPIUD in Tanzania.

## Background

Intrauterine contraceptive device (IUD) is one of the most effective methods for family planning [1]. Available evidence suggest that the timing of IUD insertion immediately postpartum has important implications on acceptance, complications and continuation of the method [1–3]. Moreover, studies have shown that the immediate postpartum period provides an ample chance for a convenient and cost-effective contraceptive option particularly in settings where women do not usually return for follow-up visits because of distance or socioeconomic reasons [1, 2].

Globally there is inconsistent data on the complications, continuation and effectiveness of postpartum IUD insertions when used immediately postpartum [4, 5] . While IUD expulsion rates are widely variable, there seems to be a consensus on the influence of mode of delivery and IUD type as important risk factors, but the role played by the insertion technique remains debatable [5]. When TCu380A is inserted within 10 min of delivery of placenta, studies indicate the expulsion rate ranging from as low as 5% to over 20% within 12 months [5–10]. Recently however, two large multi-country studies conducted by JHPiego and FIGO using the same technique of Kelly’s forceps insertion for a high fundal placement, reported low expulsion rates similar to those expected after interval IUD insertion at 6 weeks [11, 12]. There is however still no evidence of long term follow up outcomes. Moreover, the knowledge created by these and similarly published studies is limited by shorter periods of follow-up than the standard one year for most of them, the lack of standardized insertion technique, and the mix-up of IUD types.

IUD use for contraception in Tanzania is unpopular, being used by only less than 1% of women either as interval or postpartum method of contraception [13]. Since 2015 the International Federation for Gynecology and Obstetrics (FIGO) launched its institutionalization of Postpartum Intrauterine Contraceptive Device (PPIUD) initiative in Tanzania, among five other countries [12, 14, 15]. The initiative employs standardized methods in order to mitigate complications that are commonly reported to be associated with PPIUD. So far, however, the medium and long term effects of PPIUD under the initiative in Tanzania have not been reported. This is a one-year follow-up study of women who received TCu380A placement immediately after delivery under programmatic standards in order to determine method effectiveness, continuation and safety in Tanzania.

## Methods

### Study design and settings

This is a prospective cohort study of women who had TCu380A insertion within 48 h of delivery in 6 hospitals in Tanzania between 1st December 2017 and 18th April 2018. The aim was to investigate the failure rate, complications, and risk factors for one-year continuation of TCu380A when used for immediate postpartum contraception. During the recruitment period, there were 20,276 women who delivered in these 6 hospitals and counselled for postpartum family planning. Among these women, 1114 consented for postpartum IUD (PPIUD) and were inserted with TCu380A IUD under the International Federation for Gynecology and Obstetrics (FIGO) PPIUD initiative. The FIGO PPIUD initiative for Tanzania was launched in 2015 in order to institutionalize counselling and insertion of PPIUD method in a country with low uptake of Family Planning methods and IUD in particular. Under this initiative, FIGO worked in collaboration with the National Society for Obstetricians and Gynecologists and the Tanzania Midwives Association. Six high volume delivering and teaching hospitals each from a different region were selected as training centers. For each of these hospitals, 3–4 lower level satellite health facilities were purposively selected to serve as hubs for provision of Postpartum Family Planning (PPFP) health education and PPFP counseling to women during antenatal clinics. Thus, the training hospitals and the attached satellite clinics together formed a functional unit that improved knowledge on PPFP to the women, improved healthcare workers’ knowledge and skills on PPIUD counselling and insertions and created demand for PPFP. About 2000 health care workers were trained on PPFP counselling and/or PPIUD insertions and over 8000 PPIUDs were inserted by end of December 2018.

### Participant follow-up

The study event was PPIUD method continuation. All women who received PPIUD insertions under this initiative were instructed to report to the nearest Family Planning clinic on the 6th week postnatal. Routine tracking of their progress was consented by signing consent forms and exchanging mobile phone numbers for contacts. Follow-up of the 1114 clients after the 6th week was by three monthly phone calls from insertion through 12 completed months. Thus, the last phone calls were made at the beginning of the 13th month since PPIUD insertion. Women who could be reached then had their data completed. Thus, on the recruitment day on exit after PPIUD placement, socio-demographic characteristics, exposure to PPFP information, obstetric history, insertion status and written consent to participate in the follow-up were obtained. On follow-up calls, women were interviewed about the PPIUD continuation status, complications so far experienced, the duration of time they stayed with the IUD and the currently used method if PPIUD was discontinued. The estimation of duration before method discontinuation was based on client’s recalling the date of discontinuation or number of days, or months that she retained the IUD. Whenever it was considered necessary more data were obtained from family planning clinic records or the 6th week return data from the program’s database. All client information was entered in tablets and stored in the cloud server with end to end encryption.

### Data analysis

In this study the outcome variable was PPIUD discontinuation by end of one year. Data were downloaded in excel file from the server and analyzed using Statistical Product and Service Solutions software (SPSS) for Windows version 20 (IBM SPSS Statistics, Chicago, IL, USA).

**Fig. 1:**
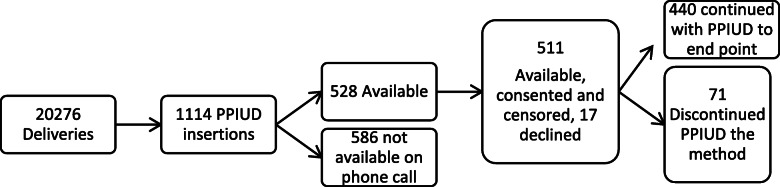
Flow chart for participant selection

We plotted Kaplan Meir curves showing the cumulative survival percentages at any time point after baseline for categories of the potential hazard factors in order to display the survival probability of the PPIUD clients by end of one year (12 months). For the variable categories, the Log Rank test was used to ascertain existence of statistically significant difference in progression rates between factor categories towards the end of one year. In order to qualify the independence of the observed effects on the hazard of retaining the IUD to one year since insertion, we subjected 10 factors into a Cox proportional hazards model if Kaplan Meir curves or clinical judgement suggested that the proportional hazards assumption for the factor was reasonable. In all statistics a *p*-value of 5% was taken as significant. We used the STROBE cohort checklist when writing our report [16]. The project received ethical approval from the National Institute for Medical Research, Tanzania (reference number NIMR/ HQ/ R.8a/Vol. IX/2016) (Fig. 1).

## Results

Of the1114 women with PPIUD, 528 (47.4%) could be reached, 17 declined interview thus leaving 511 (45.8%) who gave consent and completed the interview. The analysis of delivery information of the women who could not be reached on phone call indicated that they were not significantly different with the 528 women who were contacted in terms of mean age 25.97 ± 6.14 Vs 26.78 ± 6.39, *p* = 0.5; number of pregnancies 2.37 ± 1.64 Vs 2.35 ± 1.6, *p* = 0.8 and proportion representation of the sample within each of the 6 regions ranging from 27.3 to 35.2%. Among the 511 who could be analyzed, 440 (86.1%) still had the IUD in situ by end of one year and the IUD was discontinued in the rest 71 (13.9%). The characteristics of study participants are displayed in Table 1.

**Table 1:** Distribution of baseline characteristics by status of PPIUD continuation at 12 months

Characteristics	N (%)	PPIUD retained	PPIUD discontinued
**Age (years)**			
16–24 yrs	93 (18.2)	81 (87.1)	12 (12.9)
25–34 yrs	289 (56.6)	251 (86.9)	38 (13.1)
35 and more	129 (25.)	108 (83.7)	21 (16.3)
**Region of Residence**			
Dar es Salaam	84 (16.4)	68 (81.0)	16 (19.0)
Pwani	16 (3.1)	15 (93.8)	1 (6.2)
Dodoma	40 (7.8)	36 (90.0)	4 (10.0)
Mwanza	54 (10.6)	44 (81.5)	10 (18.5)
Mbeya	98 (19.2)	77 (78.6)	21 (21.4)
Arusha	200 (39.1)	183 (91.5)	17 (8.9)
Others	19 (3.7)	17 (89.5)	2 (10.5)
**Counselled before admission**			
Yes	366(71.6)	313 (85.5)	53 (14.5)
No	145(28.4)	127 (87.6)	18 (12.4)
**Marital status**			
Married/cohabiting	448 (87.7)	387 (86.4)	61 (13.6)
Single/divorced	63 (12.3)	53 (84.1)	10 (15.9)
**Highest Education level**			
No formal education	26 (5.1)	23 (88.5)	3 (11.5)
Primary	204 (39.9)	175 (85.8)	29 (14.2)
Secondary and above	281 (55)	242 (86.1)	39 (13.9)
**Occupation**			
Business	280 (54.8)	249 (88.2)	33 (11.8)
Employed	80 (15.7)	69 (86.2)	11 (13.8)
House wife /No formal employment	108 (21.2)	89 (82.4)	19 (17.6)
Peasant	43 (8.4)	35 (81.4)	8 (18.6)
**Mode of delivery**			
Caesarean section	163 (31.9)	147 (90.2)	16 (9.8)
Normal delivery	348 (68.1)	293 (84.2)	55 (15.8)
**Number of all Pregnancies**			
1pregnancy	119 (23.3)	99 (83.2)	20 (16.8)
2–4 pregnancy	340 (66.5)	95 (86.8)	45 (13.2)
5 or more pregnancy	52 (10.2)	46 (88.5)	6 (11.5)
**Number of Children**			
1 Child	134 (26.2)	111 (82.8)	23 (17.2)
2-4Children	364 (71.2)	317 (87.1)	47 (12.9)
5 or more Children	13 (2.5)	12 (92.3)	1 (7.7)
**Accessed PPIUD educational resources**			
3–8 resources	95 (18.6)	82 (86.3)	13 (13.7)
Less than 3 resources	416 (81.4)	358 (86.1)	58 (13.9)
**Medical/social problem since insertion?**			
No	338 (66.1)	322 (95.3)	16 (4.7)
Yes	173 (33.9)	118 (68.2)	55 (31.8)

**Table 2:** Cox-proportional hazards regression model for PPIUD continuation by 12 months

Factor	Adjusted Hazard Ratio (95%CI)	P-value
**Age (years)**		
16–24 yrs	0.33 (0.13–0.79)	0.047
25–34 yrs	0.63 (0.35–1.12)	
35 and more	1	
**Counselled before admission**		
Yes	1.0 (0.57–1.77)	0.999
No	1	
**Marital status**		
Married/cohabiting	0.97 (0.47–1.98)	0.929
Single/divorced	1	
**Highest Education level**		
Secondary and above	1	0.694
Primary	1.19 (0.69–2.04)	
No formal education	1.57 (0.46–5.39)	
**Occupation**		
Petty business/peasant	1.61 (0.78–3.31)	0.397
Salary Employment	1.26 (0.72–2.21)	
House wife /No formal employment	1	
**Mode of delivery**		
Normal		
Cesarean section	2.08 (11.17–3.70)	0.013
**Number of all Pregnancies**		
1 pregnancy	0.53 (0.13–2.12)	0.142
2–4 pregnancies	0.18 (0.30–1.12)	
5 or above	1	
**Number of Children**		
1 child	0.99 (0.268–3.68)	0.672
2–4 children	1.64 (0.32–8.32)	
5 or above	1	
**Accessed of PPIUD educational resources**		
Adequate (3–8 sources)	1	0.773
Not adequate (less than 3)	0.91 (0.48–1.72)	
**Medical/social problem since insertion (Yes/No)**	8.48 (4.83–14.89)	0.001

Among the 440 women who continued with IUD after one year, 411 (93.4%) would recommend it to people socially close, 19 (4.3) would not recommend and 10 (2.3%) did not respond. The reasons given for the 19 (4.3%) who would not recommend it were various: Irregularities of menses (*n* = 4) Abdominal pain (*n* = 3) Uncertainty about the long term outcome (*n* = 9) Others (*n* = 3).

**Fig. 2:**
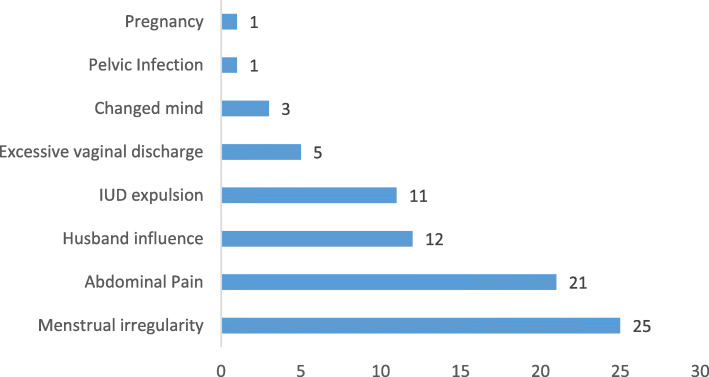
Clients reporting reasons for PPIUD discontinuation, N = 71

Among the 71 women who discontinued the method by end of one year,59 gave the reasons for discontinuation as shown in Fig. 2.

Menstrual irregularities, abdominal pain, husband influence and IUD expulsion were the most frequent reasons for method discontinuation. Pregnancy as a measure of method failure was very rare, with only one pregnancy in 511 insertions (= 2 pregnancy in 1000 insertions) although Ultrasonography done early in that pregnancy could not locate an IUD. The expulsion rate was also very low at the rate of 2.1% by end of one year.

**Fig. 3:**
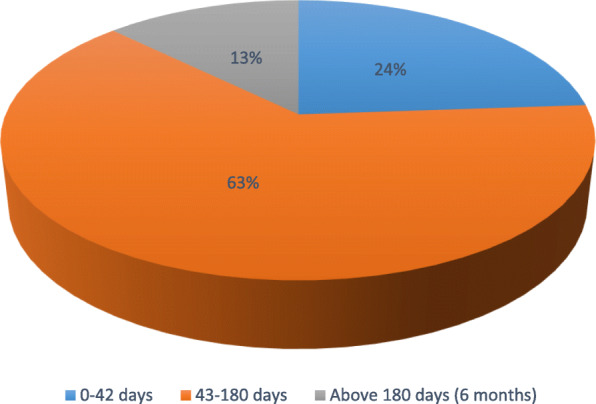
Proportion of PPIUD clients that discontinued the method in given time period, *N* = 71

The most vulnerable period for PPIUD discontinuation was between the end of puerperium and six months (i.e., 43–180 days) Fig. 3. None of the reported medical complication resulted in hospitalisation.

**Fig. 4:**
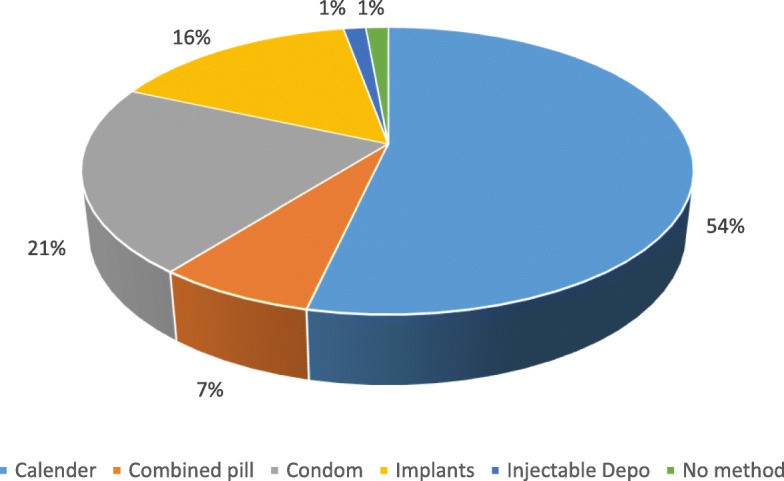
Proportion of clients using alternative method after PPIUD discontinuation, N = 71

The 71 Clients who discontinued PPIUD mostly switched to other methods as shown in Fig. 4:

Most (75%) clients resorted to more self-controlled methods of Calendar and condom but which are less effective compared to others.

### Risk factors for discontinuation of PPIUD by end of one year

**Fig. 5:**
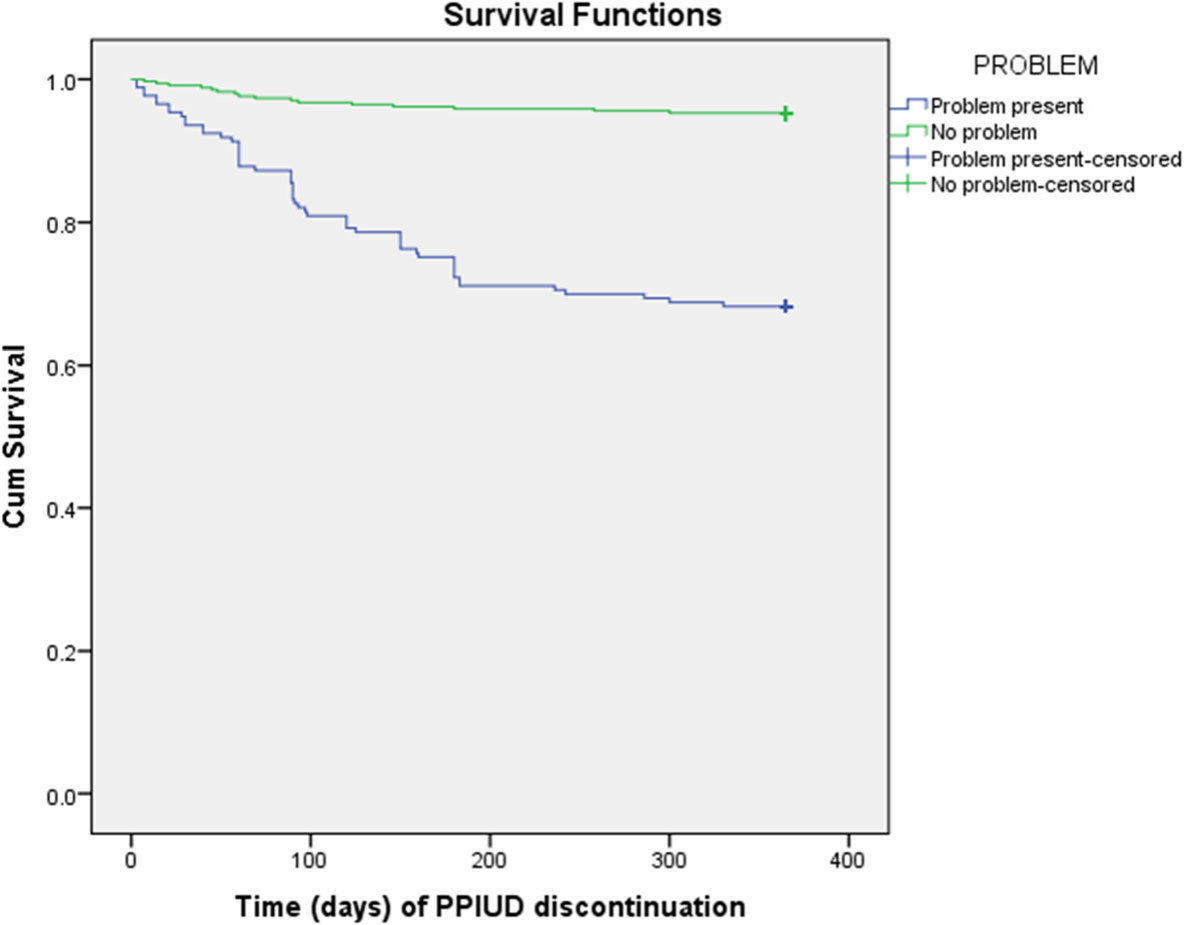
Survival probability of PPIUD clients reporting any PPIUD problem by end of 12 months

**Fig. 6:**
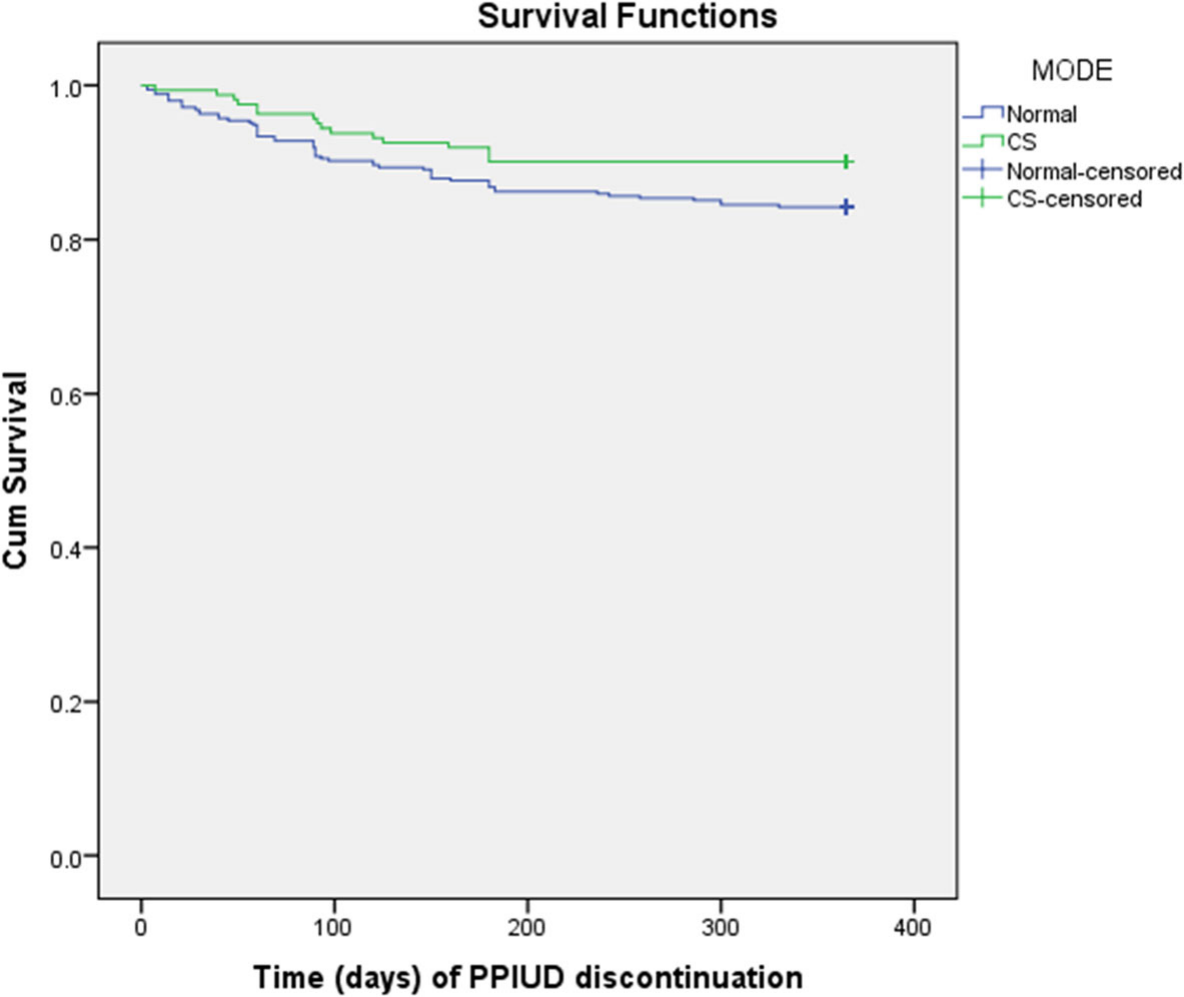
Survival probability of PPIUD clients with normal and Caesarean (CS) mode of delivery at end of 12 months

Figures 5 and 6 show the curves for reporting of any medical or social concerns (as shown in Fig. 2 and hereby labelled as PROBLEM) and mode of delivery (Labelled as MODE). Figure 5 shows a significantly difference in survival rate at 12 weeks for the clients who reported of any problem or complications and those who did not report any problem (Log Rank (Mantel-Cox) Chi square = 72.82, *p* = 0.001)*.*

Figure 6 shows significant difference in survival rate at 12 weeks for the clients who delivered normally and by Caesarean section (CS)*, Log Rank (Mantel-Cox) Chi square = 3.189, p = 0.044.*

The presence of medical or social problem attributable to PPIUD was associated with shorter continuation of PPIUD after adjusting for other risk factors. Particularly women who reported a medical or social problem had 8.47 times hazard of method discontinuation by the end of one year compared with those who did not report a problem (p = 0.001). Likewise, delivery by normal route independently increased the hazard for method discontinuation by 2.08 times relative to delivery by Cesarean section and young age particularly the youth (16–24 years) decreased the hazard for discontinuation by end of one year by over 67% relative to the older age of 35 years and more (Table 2).

## Discussion

The present study was conducted in the context of increasing utilisation of TCu380A for immediate postpartum contraception as a result of the FIGO program to institutionalize PPIUD in Tanzania but with scanty data available to demonstrate method effectiveness and safety in a long term. Tanzania is among countries with low modern contraceptive prevalence rate of only 35% for the married women among whom less than 1% are using any form of IUD [17].

This study has demonstrated that TCu380A when inserted within 48 h of delivery is effective since among the 511 women who were followed upto one year, only one became pregnant which represents a slim failure rate of about 0.2%. Moreover, for this case the IUD was not found in the uterus following an early ultrasound scan during pregnancy indicating that probably the IUD had been expelled unnoticed some time before conception. The copper IUD has long been reported to be safe and effective for contraception with failure rates of less than 1% [18, 19] although few others have reported higher failure rates particularly among the youth [4, 20].

TCu380A in current study was found to be safe, except for common minor problems reported by about a third (34%) of the women within one year. Our previous comperative analyses of women with the use of PPIUD and those without use of any method under the same FIGO program, reported comperable incidences of such complaints (data not yet published). For the current study, these complaints were predominantly in form of abdominal cramps and irregularities of menses. None of these women was hospitalized, but among them a third (31.8%) discontinued the method indicating the need to mitigate these complaints. Across literature on PPIUD it is difficult to compare the complication rates due the lack of standardized definition. If expulsion and menstrual problems were combined in current study, the complication rate would have been around 7% which is much lower than the 32.5% in a study that assessed these complications by Mishra et al. [9]. Notwithstanding how PPIUD complications are defined, many studies generally report almost similar or higher rates of complications following PPIUD [4].

Our earlier report of 2.4% expulsion rate after a 6-week followup for exclusively vaginally delivered women [21] and the current expulsion rate of 2.1% at one year follow up of a mixed sample by mode of delivery are generally better than reports by most earlier studies that often quote higher expulsion rates of up to about 40% even on shorter follow up intervals [8–10, 18, 22–25]. Although previous systematic review indicated no association of the insertion technique and expulsion rates [5, 26], the reviewed studies mostly used ring forceps, hands or other types of IUD inserters to place the IUD into the uterine cavity unlike the FIGO initiative that uses long curved Kelly’s forceps that places the IUD higher up into the uterine fundus [11, 12]. Moreover, the previous systematic reviews for the subject did not restrict the IUD type to TCu380A IUD which is considered to be superior to other types of IUD [18, 26].

Method continuation in the current study was found to be high (86%) at one year of follow-up which is comparable or better than the 55–82% commonly reported by many other published studies [3, 20, 27–31]. Moreover, the most vulnerable period for PPIUD discontinuation was that period after the puerperium to six months of delivery (i.e., 43–180 days) contrary to the period of up to 6 weeks post-delivery that is reported to be critical for IUD related complications and removal in some earlier studies [32, 33]. In connection to this, over two thirds of the women who discontinued PPIUD method and gave reasons for discontinuation, attributed it to husband influence and menstrual irregularities which could explain this timing that coincides with resumption of menstruation and coitus. Other influences of discontinuation were abdominal pain, IUD expulsion, and voluntary IUD removal in order to allow pregnancy. Generally, the overall spectrum of complications attributable to PPIUD discontinuation in current study is similar to what is commonly reported by others [20, 30].

The use of an equally effective method of contraception after the discontinuation of TCu380A was rare. Three quarters of the women who discontinued the IUD resorted to Calendar and condom methods which are among the least effective compared to other available modern methods of contraception. Non-use of an effective method after PPIUD discontinuation has been reported by others where up to half of clients did not use an alternative method [30]. This phenomenon could be a reflection of negative attitude towards modern contraception in general rather than being specific to the IUD, thus, calling for the need to intensify education on postpartum contraception and family planning in general.

Apart from the experienced problems or complications, two other important factors were identified to be independently related to method continuation including the mode of delivery and the woman’s age group. The finding that delivery by cesarean section increased the likelihood for method continuation independent of other factors supports what was observed by previous studies of reduced rate of method discontinuation when IUD is inserted at Cesarean section as compared to vaginal delivery [22, 34, 35]. This could be at least partially explained by women who have had Cesarean section being more concerned about getting pregnant again given the scarred uterus and so more likely to continue with effective contraception even in the event of side effects or the women may have been advised by health care workers that if they wish to have another pregnancy after a Cesarean section they should wait at least 24 months as uterine rupture may be less likely than after shortened periods of birth spacing. The finding that youths (16-24 years) are likely to continue with the method than older age groups (ages 35 years and above) has not been documented due to paucity of published literature comparing the continuation rates of TCu380A across age groups. Nevertheless, it is reported that youths have highest unmet need for postpartum contraception [1].

A great majority (93.4%) of women who continued with the method after one year, would recommend it to people in their close social networks. This an important step towards demand creation for postpartum family planning, especially in a setting where the rate for IUD use is less than 1% [17]. The few women who would not recommend the method to their close social networks gave several reasons for not recommending including; irregularities of menses, abdominal pain, and uncertainty about the long term outcome. This is an important feedback to the health care providers to strengthen pre and post IUD insertion counselling and education to the clients.

Although the women in this study were followed prospectively, the phone calls reported on events that occurred retrospectively, thus subjecting it to the risks of recall bias as one of its limitation. In order to mitigate this, the women had baseline data collected face to face on their first interviews and during a 6-week follow-up that could be accessed from our database and inform on some missing information. Another important limitation is a large proportion of women that dropped out of the study leaving about 46% who could be analyzed at end of one year. However, such a low yield at one year follow-up is commonly reported in case of PPIUD in developing countries [2, 31]. The long term follow-up, the use of a sample of women who underwent IUD placement using same protocol for insertion under the FIGO PPIUD initiative constitute the strength of this study and its suitability for comparison with other similar studies elsewhere in developing countries.

## Conclusions

When CuT380A is used as a method for immediate postpartum contraception under the current PPIUD model, it has a high continuation rate, effectiveness and safety. These results are important for inclusion as resource for PPIUD training, client information, advocacy and service provision.

## Data Availability

The unidentified datasets used and/or analyzed during the current study are available from the corresponding author on reasonable request.

## Abbreviations

FIGO: International Federation of Gynecology and Obstetrics
IUD: Intrauterine Device
MoHCDGEC: Ministry of Health, Community Development, Gender, Elderly and Children
MUHAS: Muhimbili University of Health and Allied Sciences
NBS: National Bureau of Statistics
NIMR: National Institute for Medical Research
PPFP: Postpartum Family Planning
PPIUD: Postpartum Intra Uterine Device
SPSS: Statistical Product and Service Solutions software
STROBE: Strengthening the reporting of observational studies in Epidemiology
TAMA: Tanzania Midwives Association
